# The Hospital. Nursing Section

**Published:** 1902-03-15

**Authors:** 


### The Hospital.

### Nursing Section.

Contributions for this Section of "THE HOSPITAL" should be addressed to the EDITOR, "THE HOSPITAL" NURSING SECTION, 28 & 29 Southampton Street, Strand, London, W.C.

No. 807.—Vol. XXXI. *SATURDAY, MARCH* 15, 1902.

### Notes on News from the Nursing World.

### PRESENTATION OF MEDALS BY THE QUEEN.

An interesting feature of the Royal visit to the West of England last week was the presentation by the Queen at Dartmouth and Devonport of medals to nurses. Directly after the ceremony of laying the founaation stone of the Royal Naval College was concluded, on Friday, three officers of the guardship *Australia* were called up on to the daïs and presented by the King with the medal for service in China. At the same time the Misses Falconer, Pinniger, and Barker, who have served in South Africa, and are now attached to the *Britannia*, had the gratification of receiving from the Queen the certificates and badges of the corps of Queen Alexandra's Naval Nursing Service. On Saturday, at the Royal Naval Barracks, Devonport, a similar presentation took place, the following nurses receiving from the Queen their certificates and badges, the Head Sister, Miss Anna French, and Sisters Annie Macpherson, Evangelina E. Harte, Sarah K. Maxwell, Ethel R. Whittington, Agnes Allsopp, Norah M. E. Johnston, Katherine M. Hickley, and Florence Belcher. The members of the service have hitherto worn the red Geneva Cross on white cloth back-ground on their right arm. The new badge has, in addition, an anchor worked in with the initials A. A. above the cross, and above that the Imperial Crown, the whole being worked in red silk and gold and silver thread, on blue cloth back-ground.

### THE QUEEN AND CORNISH NURSES.

During the visit of the King and Queen to the West her Majesty received on board the Royal yacht the nurses belonging to the Cornwall County Nursing Association, of which she is the Patroness. Thirty Queen's and village nurses travelled from all parts of the county, and, after lunching at the Royal Hotel, Devonport, proceeded on board the Royal yacht, where they were met by the Earl of Mount Edgcumbe, the president, who was accompanied by the Countess of St. Germans and the Lady Margaret Boscawen, vice-presidents. Lord Mount Edgcumbe presented Miss Michie, the superintendent, to her Majesty, and afterwards the Queen's and village nurses, with whom she graciously shook hands. In the afternoon the nurses had a good view of the launch of the battleship *Queen* and saw the first keel-plate of *King Edward VII.* laid down by the King. Before returning home tea was served, thus ending a most enjoyable day. The expenses were defrayed by the president and some of the vice-presidents of the County Nursing Association.

### THE QUESTION OF TECHNICAL QUALIFICATION.

The Workhouse Infirmary Nursing Association may be congratulated upon the interesting character of the meeting on Monday. Miss Gibson's paper was of course, the principal feature, but it will be seen from our report that in the discussion which followed many points were raised that merit attention. One speaker described as a grievance the refusal of the Local Government Board to promote to the rank of superintendent a nurse who is not technically qualified. But it is of vital importance that the technical qualification should be insisted upon, and whatever differences there may be about details, all reformers are of one opinion as to the necessity of appointing none but fully-trained nurses to the office of superintendent.

### THE NEW HOSPITAL AT LIVERPOOL.

Attached to the David Lewis Northern Hospital at Liverpool, which was opened this week, is a nurses' home with accommodation for 66 nurses. There are separate bedrooms for each, a spacious octagonal sitting-room on the ground floor, a sitting-room and library combined on the second floor, while the basement is occupied by the chapel and hospital library. There is also a school for teaching hospital cookery. In fact, the probationers at the Northern, now called the David Lewis Hospital, have always been trained as far as possible in cookery and housekeeping. The matron, Miss J. B. Anderson, has two day assistants, a home sister and a housekeeper sister, who, together with all the other sisters and staff nurses, were trained in the hospital. At present there are 200 beds in use.

### THE NURSES WHO REFUSED TO BE REVACCINATED.

It appears that Dr. Harley Brooks, the medical superintendent of Mile End Infirmary, has questioned the nurses who contracted small pox as to which they would prefer again—the risk of a very bad arm from vaccination, or another attack of small-pox. "The answer in each case," he says, "has been a preference for vaccination, even with a bad arm, and a wholesome dread of all the horrors accompanying an attack of small-pox." This, adds Dr. Brooks, is "a most triumphant victory for vaccination." So it may be; but we hope that the lesson which the nurses have learnt by sad experience will also be taken to heart by the authorities of the Mile End Infirmary, and that "the offer of re-vaccination," to which Dr. Brooks refers, will, in future, be accompanied by an intimation that it can only be refused at the peril of dismissal.

### THE SIGNIFICANCE OF UNIFORM.

An Admiral of the United States Navy has been giving an address to nurses, in the course of which he insisted upon the need of obedience to orders. "The relation of the nurse," he said, "to the physician in charge of a case, is practically the same as that of the sailor or soldier to his commanding officer, or that of the commanding officer to his nation." He did not, in selecting obedience as the prime virtue, maintain that those in authority could commit no wrong; but he contended that while obedience to orders might result in a blunder in one instance, it would result in rightful action in a thousand instances. Admiral Melville also entreated his audience to be proud of their distinguishing garb, since around it in the future would gather the memory of their own victories. "No soldier with a record," he continued, "would part with his uniform since it is associated with his manliest efforts, and in the same way the nurse entering the field should try to live up to hers, and regard it as that which some day will be a badge of proven efficiency and of tried courage."

### A LIBRARY FOR LAMBETH NURSES.

It is an ill wind that blows no one any good. The prevalence of small-pox has prompted the Lambeth Guardians to provide the nurses at the workhouse infirmary with a free library of works of light literature and books of reference. It had previously been the practice of the nurses to make use of the outside free libraries, and some of the guardians fearing that small-pox might be introduced by this means into the infirmary, urged that an alternative supply should be furnished. The nurses are naturally pleased to be able to obtain books in the building.

### "MAKE HAY WHILE THE SUN SHINES."

While the Bishop of Manchester has singled out the rage for Ping-pong as a possible cause of the increasing indifference of young girls to the higher interests of humanity, the Gateshead people have arranged a Ping-pong tournament in order to assist the funds of the Nursing Association in that town. The tournament will be held in the Town Hall, Gateshead, on Thursday next, and four prizes will be given. Whatever views may be entertained as to the merits, or otherwise, of Ping-pong, there is no reason why nursing organisations should not derive any profit they can from enthusiasm in a game that can only enjoy a passing popularity.

### DROMORE DOGGEREL.

An action for libel has been brought by a nurse in the workhouse hospital at Dromore West against the doctor of that institution. One of the allegations of the plaintiff is that when she objected to the doctor keeping his dogs in the fever hospital, he posted on her door the following verse:—

> If workhouse dogs a nuisance are,
> To find the reason you need not seek far.
> Like cats old workhouse nurses are,
> Jealous of those younger than they are.

Mr. Justice Boyd, before whom the case came in the Irish King's Bench Division, said he "did not understand a man going to a lady's door and pasting a note on it," whereupon the counsel for the defendant made the very lame answer, "It was only in reference to her age." The Judge, who was asked to remit the action to the County Court Judge at Sligo, refused, but, whatever may be the decision as to the libel itself, the whole internal discipline of workhouses of this type evidently needs the attention of the Irish Local Government Board.

### CROYDON INFIRMARY.

The Medical Officer of the Croydon Workhouse Infirmary recently complained that it is difficult to obtain the services of probationers. We can quite believe this, but a very different state of affairs prevailed some time ago. When Miss Julian was superintendent nurse it was not unusual for an applicant to wait for a vacancy for four or five months. We question whether the proposal to pay probationers a salary of £6 for the first year and furnish them with uniform, will be a sufficient attraction, so long as there is a doubt about the nature of the training and certificate, which under existing circumstances is unavoidable.

### A NEW DEPARTURE AT DEAL.

At the annual meeting of the subscribers to the Deal and Walmer Victoria Cottage Hospital it was decided to add a private nursing staff to the institution. The nursing staff of the hospital, which was opened in June, 1898, with five beds and now had twelve, consists of the matron, a staff nurse, and one probationer, a paying probationer being occasionally received. The proposal to establish a nurses' institute was not received with unmixed favour, but it was carried by 27 votes to 12. Probably some of the subscribers who went to the meeting feeling doubtful about its wisdom were influenced by the intimation that the matron stated that the institute would very much minimise her work, and not in any way interfere with her present duties. A sufficient practical experience may modify the views of the matron and the subscribers on both these points.

### NURSING AT GUERNSEY.

The matron of the Victoria Cottage Hospital at Guernsey writes to express her surprise at our statement that there is a want of trained nurses in the Island of Guernsey. We publish her letter with pleasure, but we must repeat that, in the case we mentioned, the friends of the patient, though most anxious to secure the services of a nurse, were absolutely unable to obtain them.

### THE NURSES' UNION.

Miss Dashwood, honorary secretary of the Nurses' Union, held her annual "At Home" on Thursday last at Cambridge Gate, Regent's Park. There were at least 25 different hospitals and nursing institutes represented, besides nurses working on their own account. Indoor uniform was optional; many members took their cloaks off, and the costumes were very varied. Tea was served in the dining-room, and afterwards in the drawing-room there were addresses, recitations, vocal and instrumental music, and games. Canon Girdlestone gave an address on "Burning and Shining," and Miss Dashwood spoke a few words on "Drifting," basing her remarks on the unfortunate accident which had just happened to the *Etruria*.

### SHORT ITEMS.

On board the *Lake Erie*, which arrived at Southampton on the 7th, were Nursing Sister A. J. Garlick, A.N.S.R., and M. E. Loveday, civil nurse, locally engaged, and entitled to her return passage to South Africa.—The lecture to nurses by Mr. John Poland at the City Orthopædic Hospital, on Friday, March 21, will be on "The Blood Vessels."

### Lectures on Gynæcology for Nurses.

By Robert Jardine, M.D., M.R.C.S., F.F.P. and S.G., F.R.S.E., Senior Physician to the Glasgow Maternity Hospital, Examiner in Midwifery to the University of Glasgow.

### LECTURE VII.—THE TREATMENT OF DYSMENORRHŒA.

There can be little doubt that the mode of living, dressing, etc., in vogue among girls predisposes them to disturbances connected with the menstrual functions. Special care should be taken of girls between the ages of 14 and 17 while menstruation is being established. Among the poorer classes this is the time at which a girl usually leaves school and begins to work, and it is therefore almost impossible for her to take care of herself. If she is a message girl she will be out trudging about through all kinds of weather, and often carrying loads which are far too heavy for her. If she is in a shop she will be on her feet for long hours, and too often she cannot regularly attend to the calls of nature. If the girl is in a position which does not require her to work for a living she will probably be at school working hard at her lessons, developing the brain at the expense of the body. The chances are that she will not take sufficient and proper exercise, although at the present day there is a vast improvement on this point. At this time in a girl's existence the inexorable laws of fashion rule that she must begin to encase herself in a firm corset, which necessarily increases the inter-abdominal pressure considerably and tells upon the pelvic organs which lie at the lowest part of the abdominal cavity. The thin shoes which girls usually wear are quite unsuited to protect their feet from cold and damp in our winters, and the result is that many of them suffer from cold feet. The circulation of the lower extremities is closely connected with that of the pelvic organs, and anything which interferes with the one tells on the other. Considering the way in which our girls are brought up there is small wonder that so many of them suffer so much from pelvic diseases.

As nurses you have a good deal in your power to remedy this unfavourable state of matters, by explaining to mothers the importance of taking special care of their daughters during these critical years. It is the duty of every mother or female guardian of a young girl to warn her about the change which is about to occur in her life, and to impress upon her the great importance of being specially careful not to expose herself to cold, damp, or over fatigue during her periods. These remarks are not specially intended to apply to the prevention of dysmenorrhœa, but to all disturbances of the menstrual function.

The only way to cure dysmenorrhœa is to remove the cause. It is easy enough to relieve the intense suffering by giving opiates, but this must only be resorted to under exceptional circumstances, and must not be persisted in at future periods. Some mothers are foolish enough to give their daughters alcohol, often in the form of hot toddy, to relieve their sufferings during their first few periods. This as well as the use of opiates cannot be too severely condemned, and as nurses you should set yourselves strongly against any such practices. They are fraught with extreme danger. The application of heat to the lower part of the abdomen by means of hot cloths, poultices, or a hot hip-bath will give considerable relief in some cases, and, at all events, it will not do harm.

As I have already said the cause must be treated. If it is constitutional, remedies appropriate to the disease must be administered, and the system must be toned up. When the uterus is infantile in type sometimes electricity does good, but these cases are very difficult to deal with. If the ovaries and Fallopian tubes are diseased they must be treated. If they are hopelessly diseased nothing short of their removal will cure the condition. At one time the tubes and ovaries were frequently removed for dysmenorrhœa, but at the present day this operation is not nearly so frequently done.

When the uterus is at fault the fault must be remedied. Any displacement must be corrected, and a suitable pessary inserted if necessary. In cases of narrowing of the canal the passage of a sound or one or two small dilators just before the period has a wonderful effect in relieving the pain. This is most useful in the obstructive form.

Membranous dysmenorrhœa is an exceedingly difficult form to treat. Curetting may do good, but not always. In all forms of dysmenorrhœa in married women sterility is common, but if the woman should bear a child the chances are that it will cure her unless she be suffering from the membranous form, which pregnancy does not often benefit.

The mid-menstrual pain is difficult to treat except in those cases in which it is associated with tumours, when the removal of the tumour will probably cure the condition.

*Vicarious Menstruation.*—Cases occasionally occur in which there is a periodic discharge of blood from some other part of the body than the uterus. This is known as vicarious menstruation. A few cases have been recorded in which it has occurred from the stump of the cervix after the uterus and ovaries have been removed. The flow may come from any of the mucous membranes of the body. The mucous membrane of the nose is perhaps the commonest seat of this form of menstruation. Cases have been recorded where ordinary menstruation never occurred, but the women suffered from periodic bleeding from the nose. The mucous membrane of the stomach is sometimes the source, and it has been mistaken for a gastric ulcer, to which women are so prone. It has been noticed from the intestines, from sores, from a scar, and from a nævus. Amenorrhœa proper may be present in these cases, or the vicarious discharge may occur at the same time as ordinary menstruation. Many of the reported cases have been undoubtedly in women suffering from hysteria. It is a very curious condition, and I am afraid, I must confess, that I cannot attempt to explain it.

*The Menopause.*—At the age of from 45 to 50, menstruation should normally cease. This is known as the menopause. Roughly speaking a woman's menstrual life lasts about 31 years. It is claimed that civilisation has slightly lengthened the period. At the menopause considerable changes take place in the organs of generation. The uterus becomes much smaller, that is, it atrophies. The ovaries also atrophy, and cease to discharge ova. The discharge of ovules does not, however, always cease with menstruation. Cases are not unknown in which pregnancy has occurred after the menopause, but these are rare. At the menopause many women rapidly put on fat. At the same age men often do the same, but it is probably commoner among women. If much fat accumulates about the heart serious inconvenience will be caused. Any exertion will be liable to bring on palpitation and hurried breathing. Flushes of heat are often complained of. The nervous system is often disturbed at this time. At the beginning there may be exaltation of the mental condition, but depression is much more common, especially towards the end of the menopause. Insanity is not infrequent. The woman stands a good chance to recover provided there is not a strong hereditary tendency to insanity. In some cases the menstrual flow ceases without any great irregularities, but in many there are months of irregular periods and sometimes the periods are very profuse. It must always be borne in mind that metrorrhagia at this time may not be due to the menopause, but to malignant disease. Cancer often manifests itself then.

Such a radical change takes place in a woman's system at the menopause that we cannot wonder at its being often accompanied by considerable disturbance. It is a time when the vital powers are failing, so that any latent diseases and defects are sure to make their presence felt.

*Treatment.*—The menopause of itself does not as a rule require any special treatment, but many of the symptoms which arise require attention. The flushes of heat are sometimes very disagreeable. The administration of bromides will often relieve them. If the patient tends to put on too much fat, she should regulate her diet and take plenty of exercise. In cases of serious nervous manifestations it may come to be a question of the advisability of having the patient treated in a special institution; at all events, she should be under careful supervision. When patients become depressed, if they have nothing to occupy their minds, they should be encouraged to take up some work and be kept as cheerful as possible. If any latent diseases manifest themselves they must be treated appropriately. If there is profuse metrorrhagia, and especially if it is followed by a flow of whites, a local examination should be made, for fear of malignant disease being present.

### The late Mr. William Rathbone, of Liverpool.

A GREAT PHILANTHROPIST.

It is with deep regret that we have to report the death of Mr. William Rathbone, of Greenbank, Moseley Hill, Liverpool. As the *Times* justly states, "in a quiet and unassuming way Mr. Rathbone had left his mark on the practical philanthropy of the Victorian era." For upwards of 40 years Mr. Rathbone devoted himself and his means to the improvement of nursing, and to him we owe in large measure, if not mainly, the introduction of the district nurse, whereby the poor are placed in a position to secure the services of a capable nurse in their own homes. Mr. William Rathbone was a vice-president of the Royal National Pension Fund for Nurses, in which he took the deepest personal interest from the outset. How much that fund owes to his quiet advocacy on all occasions among his own circle in the North and wherever he happened to be, few will ever know. It may be said of Mr. Rathbone that in his day and generation he set an example to the busiest and most prosperous members of society which it would be well for themselves as well as for the nation that the majority should follow. An amiable personality, a thorough man of business, a capable speaker, always sure of his facts and therefore commanding attention, the influence he wielded was infinitely greater than that of many men whose names are far better known to the public, and whose services to the nation will in no way compare with those he so self-denyingly rendered.

We take the following interesting account from the *Times*:—

"Mr. Rathbone married, in 1847, a daughter of the late Mr. S. S. Gair, of Liverpool, and the circumstances of her fatal illness in 1859 brought home to his mind the fact that untold misery must be suffered by the poor, who could have no such skilled nursing and attention as that with which she had been provided. On the death of his wife Mr. Rathbone asked the nurse who had attended her if she would undertake both the nursing of the poor in their own homes in a district of Liverpool and the giving of such elementary instruction on nursing and health subjects as she might think desirable. She consented, was supplied by Mr. Rathbone with all the needful appliances, and started work. In a few weeks, however, she begged to be relieved of the task, declaring that the misery and wretchedness she had seen in the houses of the poor were so dreadful that she could not endure them any longer; but Mr. Rathbone encouraged her to persevere in her mission, and the results of her work, both in saving the lives of the sufferers and in inculcating doctrines of health, were so great that other nurses were engaged. At that time, however, the supply of trained nurses was extremely limited, and on the suggestion of Florence Nightingale, who was consulted on the matter, a training school and home for nurses was provided by Mr. Rathbone in connection with the Liverpool Royal Infirmary with the object of supplying nurses (1) for the infirmary, (2) for attending on the sick poor in their own homes, and (3) for private families. Within four years of the time when the first nurse was sent out by Mr. Rathbone the entire city of Liverpool had been divided into 18 districts, each of which had its trained nurse for the poor and its committee of ladies to superintend her work. In 1864 a similar organisation was carried out in Manchester, and in 1868 the East London Nursing Association was formed. The Metropolitan and National Association followed in 1874 with Mr. Rathbone as a member of the council, and "district nursing" was adopted in many other parts of the country as well. It received the greatest impetus when, in 1889, the Queen Victoria Jubilee Institute was established with the £70,000 set aside for that purpose by Her Majesty out of the subscriptions raised by the women of England. Mr. Rathbone was naturally called upon to assist in the task of putting this institute on a sound footing, and the Provisional Committee, in their report of November, 1889, signed by the Duke of Westminster as chairman, said:—

'The Provisional Committee cannot close their report without placing on record their sense of the valuable services rendered by Mr. Rathbone, who undertook from the commencement the onerous duties of honorary secretary. . . . The committee has largely profited by his assistance and wide experience in similar work.'

"In 1865 Mr. Rathbone was the means, by guaranteeing for three years the cost of the services of Miss Agnes E. Jones in the Liverpool Workhouse Infirmary, of starting the further movement for introducing skilled nurses into union workhouses in place of unskilled and more or less infirm pauper women."

Miss Agnes Jones did yeoman service in the Liverpool Workhouse, and her useful life was all too speedily ended. Her example and work in Liverpool are still remembered with gratitude, and have produced results, thanks in large measure to the late Mr. Rathbone, which have made their influence felt throughout the country. It is not a little remarkable that Mr. Rathbone should have refused all public recognition of his great services to nursing, which he seems to have regarded as but the discharge of a sacred duty carrying its own reward. So long as England endures, and so long as her hospitals and nursing institutions maintain their high position for efficiency and excellence, the name of Mr. William Rathbone must endure and cannot fail to be handed down with gratitude to succeeding generations.

A Liverpool correspondent writes:—"The death of Mr. William Rathbone deprives Liverpool of the most beloved of her citizens. The task of introducing skilled nursing into the workhouses was not light, and many would have been disheartened by the difficulties met with at every turn, but in the case of Mr. Rathbone, steadily and perseveringly one by one was overcome. After securing election on the Select Vestry and suggesting the reform which was so much required, he generously undertook to bear for three years the additional expense which the new system would entail. This offer was accepted, and by the aid of trained nurses from the Nightingale Training School, under an experienced superintendent, the nursing in the workhouse was re-organised. At the end of two years the Select Vestry adopted the reformed system. There was indeed no other course open to them, to go back was impossible, to go forward the only way, and their example was soon followed by the Poor Law authorities in other large towns, until we now have admirably constituted hospitals, and in many cases excellent training schools attached to our workhouse infirmaries.

"When first Mr. Rathbone's scheme for district nursing of the poor was inaugurated the nurses lived in lodgings, as near to their districts as possible, but it was soon found to be advisable, both for the comfort of the nurses and for the further growth of the work, that homes should be founded, where seven or eight nurses might reside under the care of a trained superintendent. There are now four homes in Liverpool, the gifts of different generous friends, and 42 nurses are employed.

"In almost every large town in the United Kingdom there are found to-day these district nursing associations. Thus Mr. Rathbone saw his great dream become universal and national reality, and his labours crowned with success.

"The duties of the district nurse as understood by Mr. Rathbone do not end when the patients are attended to professionally, but to quote his own words in a letter sent to the nurses in Liverpool, only in December last: 'You are not inferior servants doing inferior work for inferior wages, but trained and skilled workers, carrying out intelligently the treatment prescribed by the doctor. There is not much danger of your forgetting that aspect of your work, but I hope that you will always bear in mind that in all nursing work—and especially in the work of nursing the sick poor—intelligence and skill are not enough, but that you are Christian ministers, *ministering* to the comfort and character of your fellow creatures.'"

### The Scarcity of Workhouse Nurses.

PAPER BY THE MATRON OF BIRMINGHAM INFIRMARY.

Miss Gibson's Views.

The meeting organised by the Workhouse Infirmary Nursing Association on Monday afternoon was well attended, the Committee Room at the Examination Hall being full. There were a good many Poor Law Guardians and others interested in Infirmary nursing, and among those who wrote to regret not being able to attend were Miss Louisa Twining, Miss Lückes, the Dowager Lady Jenner, Dr. Solomon Smith, Mr. C. S. Loch, and several officials of the Local Government Board.

Mr. William Bousfield presided. His introductory remarks were chiefly historical, recalling the formation of the Association 22 years ago. Public opinion, he remarked, had changed considerably since then; people had come to see that good nursing was essential, and the difficulty now was how to get well-trained nurses for the Infirmaries. It was a problem well worth the attention of the Local Government Board. He concluded with a sympathetic reference to the death of Mr. Rathbone, who had done so much to advance the training of nurses in the north.

Miss Gibson, matron of the Birmingham Infirmary, read the following paper on "The Scarcity of Nurses in Country Workhouses, its Cause and Cure."

With the first part of the subject, the scarcity of nurses, I don't think I can have much difficulty, and even the second part—the cause—finds at least some elements on which I fancy we may all agree, although it also probably holds some points of disagreement. But the third—the cure—who shall find that, and where does it lie? The temptation to miss the two first altogether, and go on—not to the root of the matter, that, I fear, is beyond me, or indeed beyond any one person—is very great, and the pleasure of dealing with a subject not somewhat discouraging and worn out, but full of possibility and, one hopes, of promise, is so much more keen that I find it requires a strong effort of will to begin at the beginning and to ask, Is or is not this scarcity of nurses a fact? Alas! I fear there can only be one reply to this, the supply of nurses is undoubtedly unequal to the demand. We have only to look week by week at the advertisement sheets of the nursing and Poor Law papers, to see how difficult it is found for even the medium-sized Unions to obtain nurses, while for the small ones it seems almost impossible. It is I think obvious that this lack of candidates does not arise from the dislike of nurses to enter the Poor Law service—as a service—but to the conditions under which they have to work. This view is, I think, borne out by the fact, that the large and well-equipped infirmaries, which are still under the Poor Law, but which can offer inducements of comfort and teaching, have no lack of candidates of the class whom we desire to train as nurses, and makes it clear that we have to look elsewhere for the reasons which deter candidates from answering the advertisements which are so common and so costly. In the case of the superintendent nurse, this deterring influence arises entirely, I believe, from the fact of her position and surroundings in a workhouse. The guardians are compelled to engage a trained and competent person, and then having secured the services of a well-educated, fully-equipped woman, probably fresh from hospital or large infirmary, to place her at once under the control of a master and matron who have no knowledge of her work and no sympathy with her point of view. She finds herself in an atmosphere which she has never breathed before. Alone, probably misunderstood, and almost certainly interfered with. The order certainly makes her subject to the medical officer as regards the nursing of the sick, but she is not considered capable of keeping her ward clean, or of knowing what is necessary for the comfort and care of her patients, without the intervention of the absolutely untrained—in nursing—master and matron. Is it reasonable to expect her to remain? Of course she cannot bear the isolation of her position, and she speedily goes back to her former work, or joins the immense and growing army of district and private nurses.

This I believe to be the chief reason why superintendent nurses are difficult to find, and far more difficult to keep.

But the case of assistant nurses is somewhat different, and here our more complex difficulties begin. At the present time, with the exception of the Northern Workhouse Nursing Association, I know of no organisations which exist, which give systematic training for one or two years; and the nurses of this Association, who have had good experience in a large infirmary for two years, make valuable assistant nurses. But they are comparatively very few in number, and can only fill a very small fraction of the numerous vacancies. As time goes on the number of good candidates who are willing to undergo two years' training without any definite status at the completion of their agreement with the Association will become less and less, and it must be increasingly difficult for this Association to continue even the work it is doing now. Where, then, are we to turn for suitable assistant nurses? To the army of young women who have done a little spasmodic nursing among their friends, or in their neighbourhood, or who, having been called in by a doctor to assist him in an emergency, have discovered themselves to be "born nurses" and donned a uniform? Surely not. And yet, where else?

It is obvious that intelligent women will not take up work which will not tend to improve their position, and it is this fact which renders the efforts of Guardians to replace the impossible assistant nurse by the would-be probationer, so dangerous to the profession, and so disastrous to the probationer.

A Minimum of 200 Beds.

I think it may fairly be said that no infirmary which contains less than 200 beds, and has a resident medical officer, can make any pretence of training. Indeed, personally, I should prefer to raise the number of beds to 400, because it has to be remembered that the fewer the beds the less the percentage of acute or surgical work, and it is obvious that without plenty of both no probationer can become an effective and qualified nurse. What, then, can be said or thought of the number of small infirmaries who are now advertising for probationers, offering them three years' training and a certificate, when they have no means of teaching them sufficiently well to make a certificate of any value. This practically means that the standard (if there is such a thing) of training, already low enough, is being lowered, and that a number of innocently inefficient nurses are being thrown on the profession. I do not think Guardians are to blame in this; and the Local Government Board will not, very properly, acknowledge this training, but by and by, when these probationers form the only basis of supply, will this acknowledgment at present withheld not be almost a necessity? Is this fair to the sick? Is it fair to probationers themselves? Is it fair to the nursing profession generally? I can only emphatically answer, "It is not." You will tell me this has all been said before, and so it has. I hope it may never be necessary to say it again, but I do not forget that when at a conference at Malvern in May of 1898, four years ago, I suggested that the large infirmaries might help the small, the idea seemed to be taken up, and though my meaning was somewhat misunderstood, there was some hope that something might be done. Now we are no further on than we were then.

And what is to be the cure? First let us frankly acknowledge that the present *impasse* cannot continue, that the sick

### THE SCARCITY OF WORKHOUSE NURSES.

poor must be nursed and well nursed. Let us try to put aside our personal prejudices, and work with one heart and mind not for the good of one infirmary or one board or one district, but for the good of the whole country. Some of us feel that we are doing very well, and we do not want to be disturbed in our own little corner. If any purpose is to be served, the large infirmaries must help the small; and to do this heartily and thoroughly the Local Government Board the inspectors, the Guardians, the medical officers, the matrons, and, in another way, the nurses themselves must give their best thought, and their best work, to formulating a scheme which will enable us to nurse all our poorest and most chronic cases without creating a lower grade of nurses, or bringing in an element of amateurishness, or of incompetence.

Now to prevent the deterioration of the nurses it is necessary that all should have a thorough training, and the important point to consider is, How is this to be given?

This is of the very first moment. I do not believe that it can be given in a small infirmary—that is, one of less than 200 beds—consequently these must be eliminated from our minds as schools of nursing, and must be added to those Unions who require to be helped to nurse their sick. An effort has been made—a most admirable and praiseworthy effort—to do something towards general efficiency in Yorkshire, and one feels that this is a step in the right direction, but it is partial—local, and we need something which is thorough and national.

### The Cure.

The ultimate and definite cure, the perfected scheme and plan, cannot, as I said before, emanate from one head; it must be the outcome of the accumulated wisdom of experts in medicine, in nursing, in poor law, *i.e.* of medical men, Local Government Board inspectors, Poor Law Guardians, clerks, Poor Law matrons of large and smaller infirmaries. It must be discussed dispassionately with no preconceived notions, and no prejudices, but with a clear desire and determination to sink individual interests and claims, and consider only the greatest good of the greatest number. At any rate free discussion by a committee of experts will clear the air and show what is possible to be done. I am not in a position to lay before you now a cut-and-dried plan of action, neither do I feel encouraged to do so by the fate of those which have been put forth before. They have been listened to or read, and dismissed, and when one realises that the consummation of any one of them must be a question of about half a century, it may be well as a foundation for criticism, if for no other reason, to state some of the lines on which I think it might be possible, without undue delay, to do much, if not all. I should deprecate any scheme which removed the sick from the immediate neighbourhood of their friends. No one who has not lived among the sick poor, especially the chronic sick, can realise how much the visits of their friends mean to them. Nothing in their cheerless lives can make up to them for this loss, and nothing that medical care or nursing can do will take the place or do the good of the few minutes of the breath of their own old outside life which is brought to them by their visitors. If these cases, who need nursing for their very feebleness, were left in the workhouse, they would still need skilled care, and the difficulty of getting that would be increased by the fact that every case of any interest was removed to hospital or district centre.

### Miss Gibson's Suggestions.

My own views would run somewhat on these lines. I should propose to have a Central Board connected with, or if possible, a department of, the Local Government Board. The functions of this board would be to engage probationers for a fixed period of, say four or five years, and to draft them in equal proportions to the large infirmaries, Metropolitan and Provincial, for two years. These probationers would have to be paid for, and to have a small salary and uniform during the two years of their probation. They would then be drafted off by the Central Board to a small infirmary for other two years. While there they would of course receive the salary given by the Guardians to assistant nurses. It would not be necessary for any probationer to be kept during her third and fourth years in one Union. If for any reason she was not happy, or if the Guardians did not wish to keep her, she could be changed at the discretion of the Central Board to another Union. She would be under the control of the Board of Guardians for whom she was working, and reports of her conduct and capacity would be sent quarterly to headquarters. She should undergo an examination at the hands of the medical officers and matron at the end of the first two years, and again at the end of her fourth year, before the certificate was granted. Personally, I should prefer a fifth year to be spent at one of the large infirmaries to insure that the knowledge at the time the certificate was given was up to date, but I quite see that this might lead to difficulties and complications, though I think also it would be worth doing.

Five years, or even four, is a long time for a young woman to look forward to, and I should therefore take those bound for so long at a younger age, say 21 or 22.

These probationers, when sent to the large infirmaries, should be extra to, and not part of the staff; that is to say, there should be no diminution of the usual staff on their account. Few infirmaries are so well staffed that a few more pupils would not be a great acquisition, and, of course, in most places extra accommodation would require to be built. I think, too, a woman inspector who would have to be an expert Poor Law nurse should be appointed, who would visit all the Unions in which these nurses were employed, to assure herself, and the Central Board, that their teaching and surroundings were all that was necessary, and to help and advise in difficulties which must occur.

You will ask, Would the supply of candidates for such a scheme be sufficient?—and that would entirely depend, I think, on the status given to the nurses, and the arrangements made for their comfort and happiness during their two years in a small Union.

If the Central Board had the distinction of an acknowledged Government department, and the nurses thus felt that they belonged to a respected and honoured company of workers; and if small Unions gave good salaries and good food and comfortable quarters; and if they worked under a superintendent nurse to whom they were responsible, and by whom they were taught, I believe the supply of applicants would be a sufficient one. It seems to me that some such scheme as this, carefully worked out — might afford a solution of most of our difficulties. It would not, I think, be very expensive—the expenses of the pupils in training, and of keeping together and making it possible to have full and frequent meetings of the Central Board, would be considerable, and these would be permanent expenses. But all others, additions to nurses' homes, etc., would not be very considerable, and would be final. All uniforms and salaries would, after the second year, be paid by the Boards to whom the nurses were sent as assistants. But these salaries would not be excessive, and though they ought to be good, I do not think that they ought to exceed the ordinary salaries of assistant nurses.

### The Superintendent Nurse.

A competent medical man does not attempt to remove the disease from his patient is suffering until he has found and removed the cause, and so I feel that all our efforts are lost, and our time wasted, unless we, too, seek to remove what is, if not the entire cause, at least a very large part of it—viz., the present position of the superintendent nurse towards the master and matron. The present situation is an impossible one

### THE SCARCITY OF WORKHOUSE NURSES.

—it is *most* difficult and anomalous for the master and matron, and equally so for the nurse, and it is only by an entire reconsideration and reconstruction of this aspect of the present *impasse* that any improvement can be obtained. The best class of nurse will not accept an appointment when, by the conditions by which she is bound, she knows that she may be unable to do what she believes, and has been taught to believe, to be her duty. If friction arises she suffers in repute, and in herself, and so she constantly gives up, because she cannot get what she considers right; or else she loses her first high ideals and simply allows things to drift. This last is the worst case, for she thereby loses her enthusiasm for the sick and poor, and lowers her own standard of work, and still worse, that of the young nurses who are working under her.

I do not think it would be right for me to enter into many details, while the Departmental Committee of the Local Government Board is still considering these questions, but I should just like to say that I consider that the workhouse master must be the *administrative* and *disciplinary* head of his workhouse; that any departures from the ordinary routine of the house cannot be made by any nurse or official without his knowledge and concurrence; and that he, and he alone, can deal with cases of discipline in the house or infirmary. But in the actual care of the sick, in the control and management of her subordinates, in the decision as to what linen and supplies are necessary for her patients, in the care of wards, and in the ordering of dressings, etc., the superintendent nurse should have a free hand, subject only to the correction of the medical officer or the Guardians.

I think the General Order of 1887, which makes the workhouse matron responsible for the sick should be rescinded, except in the absence of the master, when she becomes his legal representative, the sick wards should not be in any way a part of her charge. It has been suggested that workhouse matrons should be chosen from the ranks of trained nurses, and the present arrangement continued. It seems to me that this is just reversing matters and making a woman, untrained in any department of workhouse work, responsible for and chargeable with duties she does not in the least understand. I should imagine that to deal with the innumerable details, human and mechanical, which must come into the daily life of a workhouse matron, needs an experience and training which very few nurses have ever had or desired to have.

### The Late Mr. Rathbone.

I cannot conclude without referring to the loss all who are interested in Poor Law and nursing matters have sustained in the death last week of Mr. Wm. Rathbone. Only a few weeks ago he was deeply and keenly concerned about the question we have been considering to-day. To those of us who were honoured by his friendship his loss is simply irreparable, though he has left us all a magnificent legacy in the recollection of his unfailing sympathy, his tender solicitude and ready help in every form of suffering, his absolute fearlessness in the cause of right, and his perfect simplicity of aim and character. It is a great thing to have known such a man, and at the end of his beautiful and blameless and unselfish life one can only say:—

> Life's race well run,
> Life's work well done,
> Now cometh rest.

I have kept you a long time—I beg you to forgive me—and I conclude by urging you to join together to help our poor sick sisters and brothers. Let us do something, even if at first we fail. "He is not the best man nor the best General, who makes the fewest false steps—poor mediocrity may secure that—but he is the best man who wins the most splendid victories by the retrieval of mistakes. Forget mistakes, and organise victory out of them." Nothing will ever be done if we wait till we are sure.

> "Doubt will exist as long as we live in the world.
> Yet, pursuing with joy the road of virtue,
> Like the man who observes the rugged path along the precipice, we ought
> Gladly and profitably to follow it."

### The Discussion.

The reading of the paper was often applauded, and Miss Wood opened a discussion. She said she had heard a great many matrons read papers, but this was the first time she had heard a matron who looked outside her own walls. That was what everyone ought to do. The point that struck her was that, before anything could be done, amalgamation must take place. But it must not be amalgamation of the Sick, but of the Training Schools. More schools like the school at Birmingham were wanted, and more Miss Gibsons, and the large training schools should help the smaller ones. Training required a certain number of patients, a resident medical officer, and a matron. Large schools would always attract nurses, it was the small ones that found such difficulty. If some scheme could be worked out in which the nurse, whilst attached to a central organisation, could be movable, one great difficulty would be done away with. The trained nurse from a large hospital did not like the small infirmary; she either left or got into careless ways. The poor suffered in consequence of her deterioration. As to the scarcity of nurses, there was none; the profession was overstocked, and others were ready to step in. The conditions of work must be made attractive to a girl trained in a special walk of life, and then complaints would cease to be made.

The next speaker was Dr. Milsom Rowe, Chairman of the Northern Association, who said that what was wanted was better accommodation, more varied dietary, and longer holidays. The accommodation was not what it should be, and as to the diet, if it was not beef it was mutton, and if it was not mutton it was beef. There were plenty of candidates, but not the money to train them. He differed from Miss Gibson in the remark she had made about the size a hospital should be to make a good training school; he thought less depended on the number of beds than on the standard of the training; an infirmary with 200 beds was quite equal to one with 600 if the training was good. A trained nurse ought to have half a day during the week, one day a month, two hours outside the workhouse walls every day, and three weeks every year. They ought to be paid better, too. He frequently saw advertisements offering a salary of £22 to an infirmary nurse; could one get a good housemaid for that? With regard to the position of the superintendent nurse, the master must be supreme head of the building, but when that was said, all was said: the superintendent nurse should be supreme in the wards, and should control the supply of linen, etc.; the workhouses did not supply proper linen for the nurses' use. He was glad to hear the suggestion for amalgamated unions, but did people realise the enormous area that this would cover? It would mean something like 250,000 acres to each district. The thing was preposterous. And what would become of acute cases—of pneumonia, for instance—if they had to be taken 30 miles to the infirmary? Apart from the question of being removed so far from their friends there would be very little chance of recovery. Then about probationers: he thought emphatically that even a first year's nurse earned her money, and she ought to be paid something for it. He had known some very sad consequences of non-payment of nurses. Miss Gibson had spoken of five years' training; for himself he was of opinion that three years, if the training was worth anything, were quite enough. He thought the status of the nurses ought to be improved, and infirmary nurses be placed upon the same

### THE SCARCITY OF WORKHOUSE NURSES.

the level as the Queen's nurses. As to examinations, there should be six or eight examination centres all over England, to which the nurses should come and be examined by independent persons. They should bring a certificate of good conduct and good health, and no fee should be charged. He imagined that would answer the purpose, and if the Guardians would attend to the points on which he had insisted, viz., accommodation, dietary, and holidays, they would find that the supply would equal the demand.

The Rev. David Fotheringham, Chairman of the Edmonton Board of Guardians, said everyone felt when the shoe pinched, and his theory was that a scheme something like that under which certain teachers were trained for the elementary schools (they could not get into the training colleges, but were allowed to pass an examination, and had a definite prospect of promotion) would be possible, and would attract the nurses to the infirmaries. One great grievance at Edmonton was the refusal of the Local Government Board to grant a certificate even after many years' work; the superintendent nurse had recently died, and they were going to promote a nurse who had been with them 18 years; the doctor had given her a certificate, but she was not technically qualified. There was no earthly reason why the Local Government Board should insist on a resident doctor; at Edmonton there were 300 beds, and only a visiting medical officer. What did it matter whether he was resident or not? He was doing the work, and the nurses were always present at operations, and he questioned whether there could be any improvement.

Mr. Gray (Coventry), said the best thing to do was to keep hammering away till some kind of centre for the training of nurses was obtained. At Coventry their guardians had very few applicants, and some of these were by no means fully qualified, though the class of young women employed very soon proved themselves capable of becoming so.

Mr. Isherwood (Bury), thought too much was said about status. It would be absolutely impossible to displace the master and put the superintendent nurse in his position. Would she have the control of the cooking? And the supplies of linen, etc.? If so, he thought she would be in a much worse position than at the present time; there would be no real elevation. If only the various officers performed their several duties, there would be no friction. He agreed, however, that if the conditions were improved the supply would equal the demand.

Miss Haldane gave some details of how things were done in Scotland, and said that the superintendent nurses there were very much more independent than in England; they had complete control, and there was no friction.

A Gentleman from Sheffield, whose name did not transpire, was surprised that Mr. Humphreys' scheme for the removal of the sick to district hospitals had fallen so flat. Why should there not be county hospitals, just as there were county asylums?

Miss Wilson, Treasurer of the Association, urged the need for thoroughness of training. She said there was now a big profession with a strong *esprit de corps*, and the nurses must look to Miss Nightingale as their guide; she had always taught that thoroughness was the object to be aimed at. Nursing was not a question of a written certificate, but of thoroughness in ward work; a great deal of harm was done if a nurse did her work carelessly, and yet, when she came up for examination, gained a certificate. Miss Haldane had spoken of Scotland; she would like to say a word about Ireland, where, according to a letter from Lady Monteagle, nursing had recently made enormous strides. The superintendent nurses there were entirely responsible, except with respect to the control of the workhouse, and they were at any rate supreme in their sick wards. It worked well, and perhaps if England followed these examples, some of the friction might be got rid of.

### Miss Gibson's Reply.

One or two questions were asked, and then Miss Gibson replied. She said "Dr. Milsom Rowe had said there was no scarcity of nurses; did he read the advertisements in The Hospital? A Union in the West of England had advertised for six nurses and could not get them. Another infirmary had advertised three times, and had no applications." At this point a gentleman asked, "What salary was offered?" "Thirty-five pounds," replied Miss Gibson, "rising to £45." This was for a superintendent nurse. She was afraid such cases were rather common; the same Unions advertised over and over again. They would not do this if there were no scarcity of nurses. As to the number of beds requisite to make an adequate training school, Dr. Rowe appeared to be a little confused between a hospital and an infirmary. In a general hospital all the cases would be acute, whereas in even a large infirmary only a small proportion would be so. It was not a question of training in ordinary hospitals, but in infirmaries.

Then Dr. Rowe seemed to think she had suggested that the probationer should have no salary. She thought she had made it clear that that would be one of the expenses connected with a training school. Three years was quite long enough in a general way, that is, if they could get the nurses trained in a general hospital, it would be sufficient; but out of those three years, two were to be spent in an infirmary, and in order to equalise matters, and not let the standard of training be below that in a general hospital, one or two years must be added.

The infirmary with 300 beds and no resident medical officer presented an alarming situation, and nothing would induce her to undertake the position of superintendent nurse there. A resident medical officer was an absolute necessity. The non-granting of certificates was certainly a grievance. A gentlemen had asked whether the superintendent nurse should control the cooking. She should only be independent of the master and matron in respect to the sick. The general dietary would be in charge of the workhouse officials, but the superintendent should be responsible for the delicacies, and these should be obtained by direct requisition to the master. In cases of friction, people seemed to forget that the guardians were the remedy. The guardians were the head of the nurse, and of the master. One gentleman suggested that "assistant nurse" was not the proper title. She quite agreed; every nurse ought to be a probationer until she was efficient. An "assistant nurse" ought not to exist, and she had only used the expression because it was generally understood. The whole point of the paper had been missed if it was not understood that she wanted nurses to have a thorough training. Untrained nurses added to the misery and pain of the poor, even in the smallest infirmary. People sometimes talked rather scornfully about the suffering infirm. They wanted the tenderest and most gentle nursing it was possible to give them, and "I hope," Miss Gibson concluded, "that I shall live long enough to see that they get it."

The Chairman said the aim of all modern treatment of the sick was to cure them and send them out as quickly as possible. Nothing had been said about the departmental inquiry now going on in connection with the Local Government Board. He looked for a great deal from it, and he hoped that Miss Gibson's paper would be laid before it. Mr. Humphreys' suggestion would be discussed by the members appointed to conduct that inquiry, and his was a suggestion that seemed to rest upon a logical basis.

The meeting, which had lasted two hours, then broke up, with a very hearty vote of thanks to Miss Gibson for her suggestive paper.

### East London Nurses at the Mansion House.

There was a very good attendance at the annual meeting of the East London Nursing Society which was held at the Mansion House on Tuesday afternoon. The chair was to have been taken by the Lord Mayor, but in consequence of having been commanded by the King to attend at Marlborough House he was not able to be present. A very efficient substitute was, however, found in Mr. Alderman and Sheriff Bell, who, after conveying to the meeting the Lord Mayor's great regret at not being able to take the chair, proceeded to read the annual report.

The Bishop of Stepney, who moved the adoption of the report, said that a great deal was heard nowadays about the misery and squalor of the East End, and the clergy and nurses who worked there often felt that such accounts were exaggerated. But there was one cloud that was always brooding over the East End, and that was poverty, which tended to become greater and greater. It was easy to understand how quickly misery followed on the laying aside by illness of the breadwinner or toiling mother of the family. Yet sickness had its uses, for it was through sickness that the clergy and other helpers were able to reach the hearts of the poor people in the East End. The Bishop laid great stress on the indirect civilising and uplifting effects of the nurses' work. The authority that they always introduced into the homes was of great value, especially in demonstrating how authority can be combined with kindness and gentleness. Cleanliness was the next lesson taught by the nurses, which was of more value in promoting cleanly habits than all the sanitary enactments. The nurses' care for small things was then dwelt upon, and in conclusion Dr. Lang spoke of the invaluable work done by the nurses in convincing the poor people in the East End that somewhere there existed a great fund of compassion for them in their want and misery.

Mr. Alderman Edward Mann, Mayor of Stepney, in seconding the resolution, said that he was able from his own personal experience to confirm all that the Bishop of Stepney had said with respect to the Christianising influence of the nurses in the East End. He also pointed out that the society was entirely undenominational and that all who needed help received it regardless of any differences in creed.

Dr. Hadley acknowledged the debt that he and his brother practitioners in the East End owed to the nurses for their clear and careful notes of cases. They did much work that the hospitals could not do, and that the doctors could not do. They educated the poor people with whom they came in contact, and might be called the pioneers of preventive medicine in the East End. The report showed how much the work of the society had grown, and yet it had not grown sufficiently to meet the needs of the East End.

The Rev. H. C. Dimsdale, vicar of Christchurch, St. George's-in-the-East, and brother of the Lord Mayor, said that in these days people took a great interest in the poor, and many visited the great institutions and went away feeling thankful that so much was being done to relieve the suffering; but if, as they passed through the streets of private houses, they reflected that each house sheltered five or six families, they would realise that the hospitals only touched the fringe of suffering people. Mr. Dimsdale went on to say that he had ascertained that there were in Stepney 150 resident doctors and 50 more who did much work there, and that showed what a vast amount of sickness there was outside the hospitals. There were in the first place the cases that were too bad for the hospitals, such a one being that of a poor woman suffering from internal cancer, whom the hospitals could not take in, but who received two or three visits daily from the parish nurse. A nurse's life must always be a hard one, but how much more so when it is passed in the miserable, comfortless homes in the East End!

Votes of thanks having been passed to the Lord Mayor for allowing the meeting to take place at the Mansion House, and to Mr. Alderman and Sheriff Bell for taking the chair, the proceedings terminated.

### Shoreditch Nurses in Harley Street.

The twelfth annual meeting of the Association, whose nurses work in the neighbourhood of Shoreditch, Bethnal Green, and some parts of Hackney, was held on Thursday last week. This Association has lately changed its name, and is no longer known as the Haggerston and Hoxton, but as the Shoreditch and Bethnal Green District Nursing Association. Lady Jeune kindly lent her house in Harley Street, and Princess Christian was present. Many friends of the movement attended, and the nurses put in an appearance in their uniforms. Mr. Herbert Robertson, M.P., at the request of Princess Christian, acted as chairman. According to the report, which was read by the secretary, during the year the work has widely increased, especially since October, and it is now carried on by three staff nurses and seven Queen's probationers, under the skilful guidance of Miss Böge. Owing to the bazaar in the Shoreditch Town Hall, which realised £608, the debt on the Building Fund has been defrayed, leaving the institution free from liabilities, with a balance to its credit.

The Chief Rabbi, Dr. Adler, dwelt on the practical interest which had always been taken by the Royal Family in the question of nursing, and said that at the present time it might truly be said of the sick poor "and Queens shall be thy nursing mothers." There was one clause in the rules of the Association of which he was always especially glad, No. 4, which runs to the effect that the Association is undenominational, and that the nurses are forbidden to interfere with the religious opinions of the patients. And this, he affirmed, is as it should be, for suffering is unsectarian, and nursing should be the same. It was not only by their ministrations to the sick that the district nurses helped the poor. They established, as it were, schools within the home, where the inmates learnt how to help the helpless, how to cook a little for the invalid, and how to keep the house clean and in a sanitary condition. He might truly aver that the entrance of the nurse into the house meant the inauguration of a new era of happiness for the homes of the poor. And although we could not all be nurses we could all be sisters of mercy by aiding this good work, and by presenting to the Princess Christian not *arms*, as soldiers would, but *alms* as became good civilians.

The Mayors of Hackney and of Bethnal Green having addressed the meeting,

Lady Jeune alluded to the debt of gratitude which all owed to Princess Christian for having done so much to raise the tone of nursing generally, and said that she could personally bear witness to the influence a nurse is able to exercise. In her country home in Berkshire there were sad religious differences when the village nurse came. The latter, by her tact and cleverness, had done so much to pour oil on the troubled waters that now there was unity. When it was proposed to have a collection for the nursing fund of the parish, Lady Jeune said that she went herself to the Dissenting Minister, and asked if he would allow money to be collected in his chapel for the purpose He at once willingly assented, and added that he would like it to be on the same day as the Church collection.

### Presentations.

Reading Workhouse Infirmary.—Lady Wantage, on Monday last, in the name of the Executive Committee of the Workhouse Infirmary Nursing Association, presented to Miss Susannah Pinnington, superintendent nurse of the Reading Workhouse Infirmary, a silver tea-pot, bearing the inscription, "Presented to Miss S. Pennington by the Committee of the Workhouse Infirmary Nursing Association as a token of regard after 21 years' faithful work. 1880-1901."

### Death in Our Ranks.

We regret to announce the death, on March 4th, of Miss Hilda FitzRoy Wilson, assistant matron at the Royal South Hants and Southampton Hospital. Miss Wilson, who died after a short but severe illness, deeply lamented by all who knew her, was trained at the London Temperance Hospital. She held the L.O.S. certificate, and had filled various positions of trust before entering on her last post, which she held for nearly two years.

### Appointments.

[No charge is made for announcements under this head, and we are always glad to receive, and publish, appointments. But it is essential that in all cases the school of training should be given.]

Bradford Children's Hospital. — Miss Margaret Beatrice Vickers has been appointed sister. She was trained at the Royal Infirmary, Bradford, for three years and has since been sister of a female surgical ward for a year in the same institution. She has also done private nursing for a year on the staff of the Yorkshire Co-operation for Nurses at Leeds.

Clayton Hospital and Wakefield General Dispensary.—Miss Dora Pressland has been appointed matron. She was trained for three years at the London Fever Hospital, Islington, and for one year at the Cardiff Infirmary. She has since been nurse-matron of Hatfield Broad Oak Cottage Hospital, assistant matron at the Royal Infirmary, Newcastle-on-Tyne, and matron of the County Hospital, Durham. She has also done private nursing in Newcastle.

Galashiels Cottage Hospital.—Miss Helen N. Anderson has been appointed matron. She was trained at the Western Infirmary, Glasgow, and has since been nurse in St. Vigean's Combination Poor House, Arbroath, matron of Jan Charles Hospital, Grantown, and Queen's Nurse at East Wemyss, Fife.

Isolation Hospital, Douglas, Isle of Man.—Miss Eva P. Priest has been appointed nurse. She was trained at Liverpool Workhouse Hospital, and has since been charge nurse at the City Hospital North, Liverpool; parish nurse in North Wales, and night superintendent and assistant matron at the City Fever Hospital, Fazakerley, Liverpool.

Kingston-upon-Hull Workhouse Infirmary.—Miss Mina Haslitt has been appointed superintendent nurse. She was trained at Crumpsall Infirmary, Manchester, where she has since been night superintendent.

London Fever Hospital, Islington, N.—Miss Maude B. Akehurst has been appointed night sister. She was trained at the Torbay Hospital, Torquay, and General Hospital, Croydon. She has also had a year and a half's fever training at the Borough Hospital, Croydon. Since then she has been charge nurse at the Victoria Hospital, Kingston, and at the City Hospital East, Liverpool, and night superintendent at the Ham Green Hospital, Bristol.

Newton Abbot Union Hospital.—Miss Maria Kay has been appointed superintendent nurse. She was trained at the Birmingham Workhouse Infirmary, and has since been for three and a half years nurse at Bucklow Union Hospital, Knutsford, Cheshire, and for 18 months nurse at Prestwich Union Hospital, Crumpsall, Manchester.

Ormskirk Isolation Hospital.—Miss Janet Gracie has been appointed matron. She was trained for three years at the Brownlow Parish Hospital, Liverpool, and for two years at Bootle Corporation Fever Hospital. She has since been sister at Liverpool City Hospital, Fazakerley.

Royal Hospital for Sick Children, Edinburgh.—Miss Berta L. Harper has been appointed theatre sister. She was trained at Ratcliffe Infirmary, Oxford, and has since been sister in the men's surgical ward of the same institution.

Royal Portsmouth Hospital.—Miss H. F. Parsons has been appointed theatre sister. She was trained at the Royal Berkshire Hospital, Reading, where she was afterwards theatre sister.

St. Leonard's Infirmary, Shoreditch.—Miss Annie Dowbiggin has been appointed assistant matron. Miss Rosetta Emma Harris has been appointed night superintendent. Miss Dowbiggin was trained at Leeds General Infirmary, and has since been charge nurse at Park Hospital, London; theatre and casualty sister at the Royal Hospital, Portsmouth; and head sister at Mosely Hall, Birmingham. Miss Harris was trained at King's College Hospital, London, and has since been charge nurse at the Royal National Hospital, Ventnor, and ward sister at Bethnal Green Infirmary.

Toxteth Park Infirmary, Liverpool.—Miss F. A. Foulas has been appointed charge nurse. She was trained at the Southwark Infirmary, East Dulwich.

### Everybody's Opinion.

### "YELLOW" JOURNALISM EXPOSED.

Under the heading "Professional Philanthropy" the *Nursing Record* of the 8th inst., page 188, referring to Addenbrooke's Hospital, Cambridge, states: "It would be interesting to know on what ground Sir Henry Burdett extorts large sums of money from charitable institutions." The kind of extortion practised by Sir Henry Burdett is sufficiently explained by the following letter from Dr. Donald MacAlister, F.R.C.P.Lond., which reaches us as we go to press:—

Addenbrooke's Hospital, Cambridge,
March 10th, 1902.

Sir,—I learn that a casual remark of Dr. Cooper's, at the recent General Court of this Hospital, has led to an injurious misunderstanding on the part of persons who are unfamiliar with the facts and with the speaker. I refer to his assertion that "the £100 spent on Sir Henry Burdett's report" had been very ill spent. This has been taken to imply that Sir Henry received a fee of that amount for his advice to the Governors. As a matter of fact Sir Henry received no fee or honorarium of any kind. He not only placed his time and experience at the service of the Hospital, but at the end of the inquiry he made a donation of £21 to the general fund, and thereby became a Life Governor. The net cost to the Hospital of the clerical assistance employed in preparing the elaborate and instructive analyses of the accounts appended to the report was £14.

I am, etc.,
Donald MacAlister,
Chairman of the Special Committee appointed to consider Sir Henry Burdett's Report.

### DEARTH OF NURSES AT GUERNSEY.

"The Matron of the Victoria Cottage Hospital, Guernsey," writes: I was surprised to see in The Hospital of March 1st a paragraph headed "Dearth of Nurses in Guernsey," a statement which is very misleading. Like everywhere else the work varies very much; perhaps for a few months in the year a nurse might be very well employed, the remaining time she might have very little to do indeed. At present there are several nurses in the island and some have had very little work so far this year. I have had letters of inquiry from one or two nurses in England which I think most wise, as such reports often cause nurses a great deal of unnecessary expense.

### MANCHESTER SOUTHERN HOSPITAL.

The Lady Superintendent of the Manchester Southern Hospital, Manchester, writes: Will you kindly allow me to state that Miss Hannah Yardley, the new sister of our children's wards, after her training at Rotherham Hospital, was staff nurse of children's ward and night sister in that institution for 16 months; sister of male surgical wards, and of out-patients' department at the General Infirmary, Burton-on-Trent, for nine months; sister of male surgical and ophthalmic wards at the North Riding Infirmary, Middlesbrough, for six months; and private nurse on the staff of the Royal Berks Hospital, Reading, for 12 months. How I came to supply the wrong details I scarcely know; that the mistake is mine there is no doubt.

### "FRAUD AND CARELESSNESS."

"Father Raleigh, of St. Monica's Priory, Hoxton Square, N.," writes: In the issue of The Hospital, March 1st, there appears a paragraph entitled "Fraud and Carelessness." In this paragraph I beg to say that at the least there are two misstatements. The one, "She was at present engaged at Charing Cross Hospital;" the other that she obtained money from me "on the plea that she wanted clothing for herself." I think it only fair in the interests of the staff at Charing Cross Hospital to correct the first misstatement. Martha Elsie did not represent herself as being engaged at the hospital, nor did I say any such thing in my deposition. As regards the imputation of "carelessness," you would think otherwise did you know the whole history of the case. At any rate I am not alone in that distinction, but I may lay claim to a certain amount of craft in so managing the matter that I handed her over to the detective just as she was piteously asking my forgiveness.

[Our comments were founded entirely upon the case as it was reported in the daily papers, and these reports, so far as we are aware, were never contradicted.—Ed. The Hospital.]

### Echoes from the Outside World.

### Movements of Royalty.

FRIDAY and Saturday of last week were spent by the King and Queen and Princess Victoria in the West of England. They left Paddington shortly before 11 on Friday, and the train ran straight away to Dartmouth without a stop in little more than four hours. After receiving and answering a couple of addresses, the Royal party drove to the spot where the Naval College, the future home of the *Britannia* cadets, will ultimately stand. The position is a splendid one, breezy and high. The King laid the foundation-stone, and beneath it placed a set of coins and copies of current newspapers, enclosed in a casket made from timber taken from the old *Britannia.* Then the Royal party returned to the station amidst many demonstrations of loyalty. Plymouth was reached at half-past five, and the Royal visitors passed through the streets of the town receiving loyal addresses on the way, till they arrived at Devonport Dockyard, where they went on board the *Victoria and Albert* for the night. The King gave a dinner party in the evening. On Saturday morning medals and decorations were bestowed on 45 officers and 260 petty officers and men, some for service in South Africa, others for work in China. Shortly before four the ceremony of christening the new battleship took place. Queen Alexandra swung the flower-decked bottle of wine against the stern of the ship, saying, as it broke, "I name this ship the *Queen*; may God speed her, and all who may sail in her." Then she took the mallet and chisel, and with a few vigorous strokes severed the cord supporting the dogshores, the ship sprang free, and as the vessel kissed the water, the King and admirals waved their hats and lusty cheers were heard on every side. A minute or two later, upon the same platform as that on which H.M.S. the *Queen* had stood, the King, by pressing an electric button, laid the first plate of a new warship to be called *Edward VII.* The day again ended with a dinner party on board the Royal yacht. On Sunday the King, the Queen, and Princess Victoria attended service in the dockyard church in the morning, and went to tea with the Earl of Mount Edgcumbe in the afternoon. A charming incident of the Royal visit happened at Stonehouse. Just as the Royal carriage was moving away an aged lady presented the Queen with a beautiful bouquet. Her Majesty at once ordered the carriage to stop, took the hands of the aged lady in hers, and asked most kindly after her welfare. The spectators greatly appreciated the Queen's gracious act.

MONDAY being the 39th anniversary of the marriage of the King and Queen, their Majesties were the recipients of many congratulatory telegrams from various parts of the country. The Queen also received several choice bouquets. In the evening the King and Queen were present at a dinner given by Lord Farquhar, Master of the King's Household, at his residence in Grosvenor Square, in honour of the event. The bells of Windsor were rung, and a royal salute was fired in the Long Walk, in celebration of the occasion.

ON Wednesday last week the Prince of Wales, who was accompanied by the Princess, laid the first sod of the Royal Edward Dock at Bristol, which is to cost about two millions, and will not be completed for four years and a half. It is designed to hold vessels of the largest size likely to be built for a long time to come. At the ceremony, which was attended by an enormous concourse of people, the Prince made a suitable speech, in which he referred to the daring voyage of the Bristol sailor, John Cabot, who, sailing across unknown seas, brought the first tidings of the newly-discovered Western Continent.

### South Africa.

BAD news reached England on Monday from South Africa from Lord Kitchener, who in telegrams read in both Houses of Parliament announced a serious reverse of British arms, involving not only heavy losses in men and guns on our side, but the capture of Lord Methuen, who was wounded. His troops were attacked by General Delarey's force between Tweebosch and Palmietkuill, the Boers charging on three sides. Delarey's force were almost all dressed in British uniforms, which, as Lord Kitchener says, made it almost impossible for the infantry to distinguish between their own men and the enemy when the mounted men were being driven in upon them. Lord Methuen, who has a fractured thigh, is reported to be doing well.

### Foreign.

SINCE the release of Miss Stone, whose health is said to be excellent in spite of her enforced stay with brigands for six months, some particulars of her movements while she was in captivity have been published at Constantinople. She and her companion, Mdme. Zilka, were not allowed to know where they were being taken, and were always told that they were not in Bulgaria. They were, however, treated kindly, and it appears that Miss Stone will have solid compensation for her enforced detention. She has sold the story of her experiences to an American publishing firm for £2,000 and a royalty, and has also arranged to give a series of lectures in the States, for which she will receive £7,000.

### Parliament and Politics.

THE Army Estimates which have been before the House of Commons this week are of unusual interest, owing to the fact that the Secretary for War has increased the pay of the British soldier and improved the conditions of service. From the first of next month Tommy Atkins will receive "a clear shilling a day," and after two years' service he will be called upon to choose whether he will pass into the Reserve for nine years at the end of his three years' term, or whether he will re-enlist for six years more, subsequently passing into the Reserve for four years. The proposals have been freely criticised, but Mr. Brodrick has, generally speaking, been warmly congratulated upon the change as likely to prove of real value to the Army.

THERE was a disgraceful scene in the House of Commons on Monday evening. While Mr. Brodrick was reading Lord Kitchener's despatch cheers were raised by some of the Irish Nationalists, once when Lord Methuen's capture was mentioned, and again when particulars were given of the capture of prisoners and the loss of baggage and guns. One member clapped his hands in glee, and many of the Ministerialists cried "Shame, shame." When the list of dead and wounded was read out even the Irish members were silent.

LORD ROSEBERY delivered a long speech at Glasgow on Monday night, and disclaimed all designs on the leadership of the Liberal party. He cited the behaviour of some of the Irish members in the House that afternoon in support of the contention that the Nationalists could never be trusted with the control of an independent Parliament in Dublin.

### Society.

MISS ALICE ROOSEVELT, who has been somewhat conspicuous in connection with the reception of Prince Henry of Prussia in the States, is not coming to the Coronation. The President became dubious as to the desirability of allowing her to attend the ceremony when her status became the subject of international discussion. His final decision was arrived at when he found that the German Emperor and Empress would take the opportunity of her stay in London to invite her to Germany. Of course, he appreciates such courtesies, but he thinks that his daughter is at present too young to be called upon to meet them.

AN interesting exhibition opened at the end of last week for a few days at Lowther Lodge, the residence of the Hon. Mrs. Lowther. Princess Henry of Battenberg contributed a "View of Rosenau;" Princess Victoria of Schleswig Holstein some clever drawings of flowers; and the work shown by the members of the Royal Amateur Art Society in competition for its medal was exceptionally good. The needlework, especially Lady Hood's altar cloth, was very beautiful. A certain number of professional artists kindly gave drawings to be sold for the fund, and the result of the sales and any other receipts will be divided as before between the Parochial Mission Women Fund, and the East London Nursing Society. On Sunday, when admission was restricted to season-ticket holders, the Princess of Wales visited the exhibition and signed her name in a volume of autographs bought by Mrs. Morgan Richards.

### For Reading to the Sick.

### THE LIFE WITHIN.

If we with earnest effort could succeed
To make our life one long connected Prayer,
As lives of some perhaps have been and are ;—
If,—never leaving Thee,—we had no need
Our wandering spirits back again to lead
Into Thy presence, but continued there,
Like angels standing on the highest stair
Of the sapphire throne,—this were to pray indeed!
But if distractions manifold prevail,
And if in this we must confess we fail,
Grant us to keep at least a prompt desire,
Continual readiness for Prayer and Praise—
An altar heaped and waiting to take fire
With the least spark, and leap into a blaze!

*Trench.*

Come Thou, O come ;
Joy in life's narrow path,
Hope in the hour of death,
Come, Blessed Spirit, come:
Lead Thou us tenderly,
Till we shall find with Thee
Our home.

*Rev. Gerard Moultrie.*

To man, so great is his dignity, there are two spheres of action ; the one touches all external nature, and the myriad methods and degrees of intercourse between himself and his fellow man. There is another sphere, a world where mortal presences can scarcely enter, where mortal utterances sink and die ; no bustling crowds confuse its stately pageants, no blackening clouds obscure its vivid skies. Voices sound there, but they are clear and heavenly ; faces smile there, but radiant with the light of God. There is an inner life—bright, real, tranquil, to the true of heart. In silence we drink our deepest draughts of joy ; in silence we feel the real blessedness of peace.

Endeavour to be increasingly faithful to the serious, the blessed duties of the interior life. Depend upon it, it is face to face with God, at regulated times, faithfully adhered to, in prayer, in meditation, in examination of conscience, that we are not only surest of God's existence, but of the needed consolation of His loving care. "The secret of the Lord is amongst them that fear Him." In silent hours, amidst a heavenly splendour, there comes a voice—clear, beautiful ; it wakes up many sorrows, it stirs a pure regret, but it carries messages of hope that cheer our inmost being. It is the voice of God "speak, Lord, Thy servant heareth." "Come," it says, "come, learn of me, for I am meek and lowly of heart, and you shall find rest unto your souls."—*Knox Little.*

Father of all, to Thee
We breathe unuttered fears,
Deep hidden in our souls
That have no voice but tears ;
Take Thou our hand, and through the wild
Lead gently on each trustful child.

Father of all, may we
In praise our tongue employ,
When gladness fills the soul
With deep and hallowed joy ;
In storm, in calm, give us to see
The path of peace that leads to Thee.

*Julian.*

### Notes and Queries.

**The Editor is always willing to answer in this column, without any fee, all reasonable questions, as soon as possible.**
**But the following rules must be carefully observed :—**
**1. Every communication must be accompanied by the name and address of the writer.**
**2. The question must always bear upon nursing, directly or indirectly.**
**If an answer is required by letter a fee of half-a-crown must be enclosed with the note containing the inquiry, and we cannot undertake to forward letters addressed to correspondents making inquiries. It is therefore requested that our readers will not enclose either a stamp or a stamped envelope.**

*Treatment by Light.*

(209) 1. Will you oblige me with the address of a hospital in Manchester where patients can have the treatment by light? 2. Is there any hospital or home in London where a dipsomaniac patient could be cured?—*E. L. B.*

1. You would most likely find this out at the Manchester and Salford Hospital for Skin Diseases, Quay Street. 2. See "Burdett's Hospitals and Charities" under heading "Homes for Inebriates."

*Mentally Weak.*

(210) 1. Will you kindly tell me if there is any home where a poor woman, recently discharged from Hanwell Asylum, could be placed for a small fee? She is not fit to take charge of her house and family. 2. Would it be necessary to have a medical certificate in order to send her away?—*Nurse C.*

1. The Secretary, the Aftercare Association for Poor Persons Discharged from Asylums, Church House, Dean's Yard, Westminster, S.W., could advise you. 2. No.

*Mental Nursing.*

(211) Will you kindly tell me if there are any openings for mental nurses in South Africa, or in New York if the salaries are higher?—*Nurse Louie.*

Where salaries are higher, living expenses are also higher. No one, especially nurses, ought to go abroad without definite work or friends to go to. You will find lists of the chief lunatic asylums in South Africa and America in "Burdett's Hospitals and Charities." Apply for information to the Medical Superintendents.

*Midwives' Act.*

(212) Do you think that the Bill now before Parliament prohibiting uncertificated midwives will become law? and if it does, what qualifications must a midwife possess in order to be allowed to follow her calling?—*E. H.*

A midwife qualified now by training at any of the lying-in hospitals, or by the certificate of the London Obstetrical Society will certainly be recognised as qualified under the Bill should it become law.

*Resident Patient.*

(213) I am a trained nurse experienced in fevers and especially in heart cases. I share a country cottage with my sister, and I want to know if I could utilise my knowledge of nursing by undertaking the care of a resident patient, and if so how could I obtain one?—*Nurse A.*

You might write to the various medical men whose patients you have nursed and ask them to recommend you ; and you could also advertise repeatedly in a medical journal until you get what you want.

*Chartered Nurses' Association.*

(214) I am a fully-trained nurse and I should like to join he British Chartered Nurses' Association, but am not sure what qualifications are required. Can you kindly give me any information?—*A. S.*

Do you mean the Society of Chartered Nurses, 24 Princes Street, Cavendish Square, W.? If so, the secretary will give you the information you need.

**Standard Books of Reference.**

**"The Nursing Profession: How and Where to Train." 2s. net; post free 2s. 4d.**
**"Burdett's Official Nursing Directory." 3s. net; post free, 3s. 4d.**
**"Burdett's Hospitals and Charities." 5s.**
**"The Nurses' Dictionary of Medical Terms." 2s.**
**"Burdett's Series of Nursing Text-Books." 1s. each.**
**"A Handbook for Nurses." (Illustrated). 5s.**
**"Nursing: Its Theory and Practice." New Edition. 3s. 6d.**
**"Helps in Sickness and to Health." Fifteenth Thousand. 5s.**
**"The Physiological Feeding of Infants." 1s.**
**"The Physiological Nursery Chart." 1s.; post free, 1s. 3d.**
**"Hospital Expenditure: The Commissariat." 2s. 6d.**
**All these are published by the Scientific Press, Ltd., and may be obtained through any bookseller or direct from the publishers 28 and 29 Southampton Street, London, W.C.**

